# Difficult diagnosis and genetic analysis of fibrodysplasia ossificans progressiva: a case report

**DOI:** 10.1186/s12881-018-0543-7

**Published:** 2018-02-27

**Authors:** Shengjie Tian, Jianhua Zhu, Yaogang Lu

**Affiliations:** Department of Emergency Traumatic Surgery, Shanghai University of Medicine & Health Sciences Affiliated Zhoupu Hospital, Shanghai, People’s Republic of China

**Keywords:** Fibrodysplasia ossificans progressive, DNA sequence, Case report

## Abstract

**Background:**

Fibrodysplasia ossificans progressiva (FOP), an ultra-rare and disabling genetic disorder of skeletal malformations and progressive heterotopic ossification, is caused by heterozygous activating mutations in activin A receptor, type I/activin-like kinase 2 (ACVR1/ALK2). The rarity of the disease makes it common to make a misdiagnosis and cause mismanagement.

**Case presentation:**

We reported a case of a sixteen-year-old male patient who had suffered from pain and swelling in the biopsy site for two months. His physical examination presented serious stiffness and multiple bony masses in the body, with his bilateral halluces characterized by hallux valgus deformity and macrodactyly. Imaging examinations showed widespread heterotopic ossification. All laboratory blood tests were normal except for the one on alkaline phosphatase. A de novo heterozygous mutation (c.617G > A; p.R206H) were found in the ACVR1/ALK2 using gene sequencing.

**Conclusion:**

Even though FOP is a rare disorder of genetic origin, which is generally misdiagnosed, the genetic analysis could provide definitive confirmation of the disease. Awareness of such an important approach can help clinicians to avoid the commonly practiced misdiagnosis and mismanagement of the rare disease.

## Background

Fibrodysplasia ossificans progressiva (FOP; MIM #135100) is an extreme-rare and disabling autosomal dominant disorder characterized by congenital malformation of the great toes and progressive heterotopic ossification [1–4]. In FOP, the progressive heterotopic ossification, which begins in the first decade of life, is episodic, resulting from flare-ups that occur spontaneously or secondary to trauma [5, 6]. The bone formation leads subsequently to a severe ankylosis of the spine, limbs and jaw, with an unpredictable progression of the disability and mortality from cardiorespiratory complications around the fourth decade of life [3, 7]. Because of the rarity of FOP, most patients are misdiagnosed to have unnecessary biopsies performed, and to undergo intramuscular injections or surgical excisions of ectopic bone, which can trigger episodes of explosive heterotopic ossification [6, 8, 9]. Nonetheless, it has recently been shown that FOP is caused by heterozygous activating mutations in activin A receptor, type I/activin-like kinase 2 (ACVR1/ALK2), which is a bone morphogenetic protein (BMP) type I receptor [10–12]. Based on these findings, DNA sequence analysis of ACVR1/ALK2 of suspected patients can confirm the diagnosis of FOP, thus avoiding misdiagnosis and mismanagement [13]. In the current case report, we managed a sixteen-year-old male patient with the classic features of FOP by making a genetic analysis so that we presented a deep insight into the diagnosis of this rare disorder.

## Case presentation

The current case report was presented in accordance with the CARE guidelines. A sixteen-year-old male patient was brought to our emergency room with the complaints of pain and swelling in his upper back, in which a biopsy had been performed two months before. At the age of 9, the patient developed tender stiffness of his shoulders and neck. Over the next 4 years, he experienced multiple similar swellings on the upper back; subsequently, the movement of the shoulders and neck was limited, and masses grew progressively. The patient went to the specialists at some local hospitals for examination and treatment, and they managed the patient without making a confirmative diagnosis. Two months before, one of these doctors arranged a biopsy for the patient, but the results of the pathological examination only revealed that the mass was heterotopic ossification, the cause of which was unclear.

**Fig. 1:**
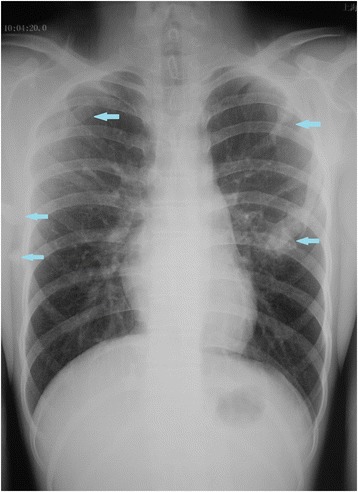
Chest radiograph showing widespread heterotopic ossification along the chest wall surrounding the scapulae (arrows)

**Fig. 2:**
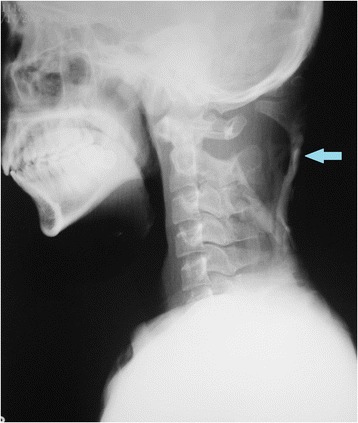
CT scan showing several initial heterotopic ossification lesions in the pectoralis major and serratus anterior (arrows)

**Fig. 3:**
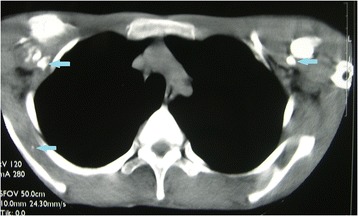
Lateral radiograph of the cervical spine showing the formation of bony bridge in the nuchal ligament (arrows)

**Fig. 4:**
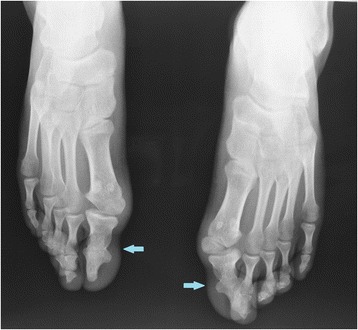
Dorso-plantar radiograph of bilateral feet showing hallux valgus deformity with macrodactyly and fusion of the halluces’ interphalangeal joint (arrows)

**Fig. 5:**
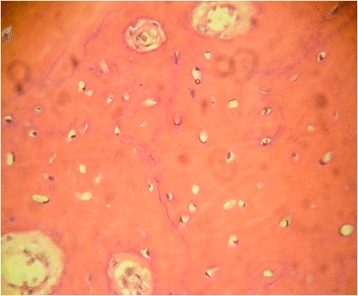
The biopsy section showing the features of inflammatory fibroproliferative and osteogenic neoplasm

The physical examination presented serious stiffness with a tiny range of motion in the patient’s neck and shoulders; multiple bony masses with irregular sizes on the neck, back and buttocks; bilateral halluces characterized by hallux valgus deformity with macrodactyly, which was more serious on the left side; and fused interphalangeal joints of both halluces. The biopsy site was marked tenderness and swelling. The imaging examinations showed widespread heterotopic ossification in the cervical spine, shoulder girdles and thorax (Fig. 1). As indicated by the radiograph of the cervical spine, the bony bridge formation was visible in the nuchal ligament, which may have predominantly contributed to the neck ankylosis (Fig. 2). The computerized tomography (CT) scan found several initial heterotopic ossification lesions in the pectoralis major and serratus anterior (Fig. 3). Dorso-plantar radiograph indicated that the bilateral feet developed characteristic clinical features of FOP: hallux valgus and fusion of the halluces’ interphalangeal joint (Fig. 4). As indicated by the laboratory blood tests, alkaline phosphatase was 276 (45–125) U/L, while serum calcium, phosphorus, hemoglobin, erythrocyte sedimentation rate, C-reactive protein, liver enzymes, urea, creatinine and parathyroid hormone were all within normal limits. From the other hospital, the biopsy section revealed the features of inflammatory fibroproliferative and osteogenic neoplasm (Fig. 5).

**Fig. 6:**
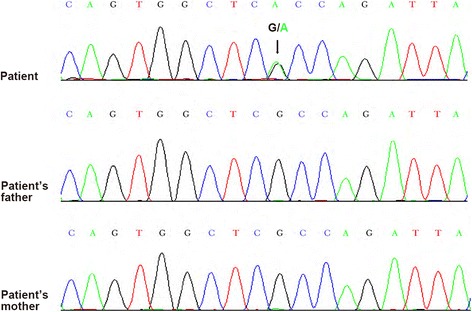
The patient’s DNA sequencing of the ACVR1/ALK2 demonstrating a heterozygous mutation (c.617G > A; p.R206H) (arrows). The DNA sequencing of the patient’s parents without the mutation

Based on the patient’s history and the results of the examinations and tests, we considered FOP as a possible diagnosis. Therefore, we collected the patient’s and his parents’ blood samples for the DNA sequence analysis of ACVR1/ALK2. The gene sequencing showed that the patient had a de novo heterozygous mutation (c.617G > A; p.R206H) in ACVR1/ALK2, which was however not detected in his parents (Fig. 6). Consequently, the diagnosis FOP was confirmed when the canonical ACVR1/ALK2 c.617G > A (p.R206H) mutation was detected in the patient.

We administered a co-therapy of a high dose of glucocorticoids (methylprednisolone, 500 mg/d for 3 days) and cyclo-oxygenase-2 inhibitors (celecoxib 200 mg/d) to relieve pain and swelling in the biopsy site. Furthermore, we advised the patient to avoid trauma in daily activities. At the follow-up one month later, we found that the pain had been relieved, but the biopsy site was still swollen.

## Discussion and conclusion

FOP is one of the rarest diseases with a worldwide prevalence of approximately 1:2,000,000, and without any racial or geographic preponderance [1, 14]. Considering the size of Chinese populations, we could estimate the incidence of at least 650 patients in China [5, 15], but only a small number of FOP cases have been reported from China [16, 17]. Actually, the vast majority of Chinese medical workers know little about the rare disease and lacked clinical experiences in diagnosing FOP so that many FOP patients were misdiagnosed or underwent unnecessary diagnostic biopsies [8], just as indicated by the current case report. It is important that a growing number of clinicians know about FOP. If the patient is observed to have developed malformed great toes and heterotopic ossification, the physician should make a genetic analysis rather than conduct a biopsy [9, 10].

In 2006, Shore et al. succeeded in locating the causative gene of FOP on chromosome 2q23-24 by genome-wide linkage analysis, and recognized the same heterozygous mutation (c.617G > A; p.R206H) in the glycine-serine (GS) activation domain of ACVR1/ALK2 which encodes a type I BMP transmembrane receptor [18]. In a normal individual, AVCR1 receptor binds an antagonist of BMP, and a muscle cell or a fibroblast does not differentiate into a bone or cartilage cell. In a FOP patient, however, the “gain of function” mutation of AVCR1 receptor makes it capable to bind BMP molecules, thereby opening BMP signal Smad pathway, increasing BMP signal inhibition, causing BMP mRNA and protein overexpression, and finally inducing the cell differentiation [19].

The management of FOP is fundamentally characterized by prevention, which includes the avoidance of trauma, deep intramuscular injections, invasive biopsies, and excision procedures for heterotopic masses [6, 20]. Up to now, however, no medical treatment is available to alter the natural history of the disease [1, 21]. High dose glucocorticoids, nonsteroidal anti-inflammatory medications, cyclo-oxygenase-2 inhibitors, leukotriene inhibitors and mast cell stabilizers have been reported to exert the effect of limited relief on chronic pain and inflammatory flare-ups [20, 22]. While definitive treatments are not yet available, the recent studies on the FOP gene and the pathophysiology of ACVR1/ALK2-mediated heterotopic ossification have documented novel approaches to the prevention and treatment of FOP [11, 23, 24]. Typically, FOP has such features as malformed great toes and heterotopic ossification. Because of its rarity, nevertheless, FOP is often misdiagnosed, as further indicated by the current case report in which the patient had received a misdiagnosis and undergone improper treatment until a genetic analysis was made to confirm the diagnosis of FOP. It is imperative that physicians have a better understanding of this extremely rare disease and be aware of an important role the genetic analysis plays in the diagnosis of the disease so that they can manage the disorder properly.

## Abbreviations

ACVR1: Activin A receptor, type I
ALK2: Activin-like kinase 2
BMP: Bone morphogenetic protein
CT: Computerized tomography
FOP: Fibrodysplasia ossificans progressiva
GS: Glycine-serine
